# Experimental mutation-accumulation on the X chromosome of *Drosophila melanogaster *reveals stronger selection on males than females

**DOI:** 10.1186/1471-2148-11-156

**Published:** 2011-06-06

**Authors:** Martin A Mallet, Jessica M Bouchard, Christopher M Kimber, Adam K Chippindale

**Affiliations:** 1 Department of Biology, Queen's University, Kingston, ON, Canada

## Abstract

**Background:**

Sex differences in the magnitude or direction of mutational effect may be important to a variety of population processes, shaping the mutation load and affecting the cost of sex itself. These differences are expected to be greatest after sexual maturity. Mutation-accumulation (MA) experiments provide the most direct way to examine the consequences of new mutations, but most studies have focused on juvenile viability without regard to sex, and on autosomes rather than sex chromosomes; both adult fitness and X-linkage have been little studied. We therefore investigated the effects of 50 generations of X-chromosome mutation accumulation on the fitness of males and females derived from an outbred population of *Drosophila melanogaster*.

**Results:**

Fitness declined rapidly in both sexes as a result of MA, but adult males showed markedly greater fitness loss relative to their controls compared to females expressing identical genotypes, even when females were made homozygous for the X. We estimate that these mutations are partially additive (h ~ 0.3) in females. In addition, the majority of new mutations appear to harm both males and females.

**Conclusions:**

Our data helps fill a gap in our understanding of the consequences of sexual selection for genetic load, and suggests that stronger selection on males may indeed purge deleterious mutations affecting female fitness.

## Background

Understanding the properties of new mutations is critical to a broad range of evolutionary theory, including models relating to the maintenance of genetic variation in the face of selection [1-3], the persistence of small populations [4], and the advantages of sexual reproduction [5-7]. Accordingly, spontaneous mutation has been the focus of numerous experimental studies (reviewed in [3,8-11]) particularly with the fruit fly, *Drosophila melanogaster*. Mutation-accumulation (MA) experiments, in which new mutations are allowed to fix by removing selection, have typically measured changes in juvenile viability (egg-to-adult survival) as an indicator of total fitness. Adult survival and reproductive success (adult fitness) will often be important contributors to total fitness, yet have been much less studied in MA experiments.

Adult fitness is important to our understanding of mutation for several reasons. For many populations, it is thought that sexual selection is a stronger force than viability selection [12]. Moreover, variation in juvenile growth, rather than survival, can have carryover effects to adult size, condition and the realization of adult fitness. This would imply that mutation pressure on total fitness could be much greater than studies examining juvenile fitness alone would imply. Because reproductively mature individuals typically express the most pronounced sex-differences in phenotype, implying divergence in selection pressures, the consequences of new mutations could be sex-specific. On the other hand, the expression of sexually selected traits may still share a common genetic basis between the sexes, as their expression is thought to depend on the overall health and vigor of the individual (the genic capture hypothesis) [13,14]. Thus, sexual selection on males could yield a correlated response in female fitness.

Indeed, sex differences in the impact of mutation have been shown to potentially shape the deleterious mutation load, with important consequences. For example, stronger selection on males is expected to improve the mean fitness of females for a given mutation rate, provided mutations have the same directional effect on fitness in each sex [15]. Thus, a reduction in mutation load due to sexual selection may reduce the cost of sexual reproduction and the severity of inbreeding depression. However, the degree to which new mutations have concordant effects is unknown, and several recent studies have demonstrated the existence of alleles with opposite effects on adult fitness in each sex. These sexually antagonistic genes may actually create a cost of sexual selection for females [16-18]. Whether sexual selection improves or degrades the mean fitness of females depends on the properties of new mutations as well as on the concordance of mutational effects between the sexes.

A few studies have attempted to measure the effects of MA on components of adult fitness in *Drosophila melanogaster*, with conflicting results. One study [19], using MA lines derived from repeated brother-sister mating, examined female fecundity under non-competitive conditions and found, surprisingly, that it increased on average when compared to a large random-mating isogenic stock. Others [20] found that female fecundity declined with MA but found no significant effect on male mating ability, probably due to a small sample size; this study also suffered from a lack of concurrently measured controls. Two other studies either found very small negative effects on female fecundity after 30 generations of MA [21], or deleterious effects on both male mating ability and female fecundity after 30 generations of MA with stronger effects on male mating ability than female fecundity [22]. Given the lack of consensus from these studies, further experimentation is clearly warranted. In addition to estimating the magnitude of selection on males and females, estimating the extent to which mutations have similar directional effects on each sex is also of interest.

MA experiments in *Drosophila *have typically been performed on the autosomes, and most frequently on the second chromosome [8]. Very few studies have explicitly examined the X chromosome [23,24], despite it accounting for around 20% of the total gene content, and none of these studies measured adult fitness. The X chromosome has a number of distinctive features particularly relevant in the context of the study of adult fitness. First, males are functionally homozygous for the X chromosome. We therefore expect selection to act more efficiently when mutations are expressed in males, the result of which could be reduced genetic load on the X. Second, the X chromosome appears to be dimorphic in terms of expression pattern, containing a relative paucity of genes with male-biased expression and an excess of genes with female-biased expression [25]. This might lead to the expectation that the fitness consequences of MA on the X chromosome are greater for females. Third, the X chromosome is predicted to be the genomic location most likely to harbour sexually antagonistic alleles. This is due to its expression pattern, with recessive male-benefit alleles being sheltered from selection in females and partially dominant female-benefit mutations enjoying the advantage of being expressed in females two-thirds of the time [26], but see [27]. This latter prediction was tested in one population of *Drosophila *by measuring the intersexual correlation for adult fitness across a sample of X chromosomes. A significant negative correlation indicated that X-chromosomes favored in females were disfavored in males, and vice-versa, and that X is a major contributor to the negative intersexual correlation for adult fitness reported in a genome-wide assay [28].

The way in which mutation and selection interact to shape the genetic load of populations for the X chromosome is unclear. On one hand, the greater effectiveness of selection on males due to the hemizygous expression of the X, which may be further reinforced by sexual selection, is expected to lower the mutation load for females at shared loci. On the other hand, the presence of widespread intralocus sexual conflict would impose a net cost to females. The overall tendency for new mutations to cause sexually concordant effects, sex-independent effects, or sexually antagonistic effects will therefore determine whether the X chromosome is a liability or an asset to female fitness. Allowing new mutations to accumulate, and determining their average effect in each sex, is the best way to ascertain the overall mutational character of the X chromosome.

We therefore sought to quantify the effects of MA on the adult fitness of males and females in a laboratory-adapted population of *Drosophila melanogaster*. The *Ives *(*IV*) population has been maintained as a large population on a fixed culture protocol for several decades and is therefore likely to be at mutation-selection balance. This population's stable environment also defines the relevant selective environment in which to measure fitness for both sexes. These features, combined with the inherent advantages of *Drosophila *as a model system, make the *IV *population an attractive study system to study the mutational process, as many of the simplifying assumptions used in models of mutation likely hold.

We carried out a MA experiment on a genetically variable sample of X chromosomes from the *IV *population. After 50 generations of MA we expressed these chromosomes, along with their controls, in males and females. For females we expressed the MA chromosomes in both the heterozygous state to mimic the normal condition of expression for new mutations in an outbreeding population, and in the homozygous state to directly compare the strength of selection to hemizygous males. We found that the magnitude of mutational effects was higher in males than in females. In addition, the intersexual correlation for fitness in the MA lines was positive, suggesting that females may indeed benefit from stronger selection in males.

## Results

### Declines in fitness due to mutation-accumulation

Nineteen X-chromosome MA lines, along with a set of Control lines, were expressed in both sexes and assayed for fitness. X-chromosomes subjected to mutation-accumulation were less fit than their controls when expressed in both sexes. Based on analysis of line means, vials containing females expressing homozygous MA-X chromosomes had 4.10 red-eyed offspring, on average (95% CI = (3.89, 4.32)), whereas vials from C lines contained an average of 5.34 red-eyed offspring (95% CI = (5.09, 5.60)). Vials with males with the same MA-X chromosomes contained 8.94 red-eyed offspring on average (95% CI = (8.41, 9.51)): vials with males from the C lines contained 13.35 red-eyed offspring (95% CI = (12.66, 14.11).

**Figure 1:**
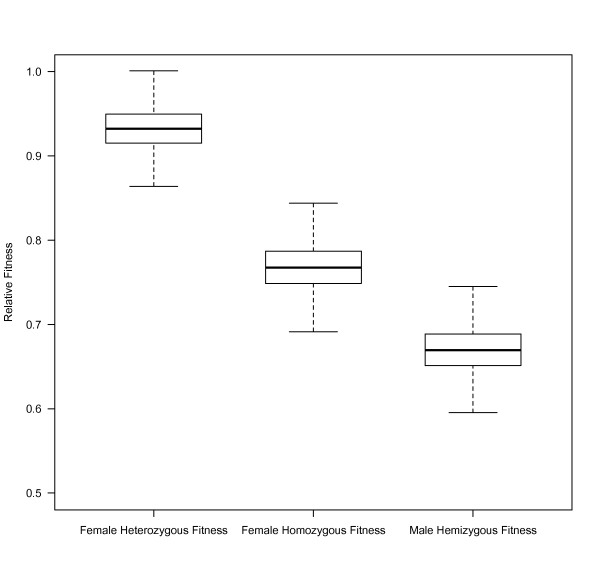
**Decline in relative fitness with MA**. Heterozygous MA females experienced the smallest decline in fitness relative to their controls, followed by homozygous females and hemizygous males. Boxes span the interquartile range, and the whiskers extend 1.5 times this distance from the box.

In terms of relative fitness, MA females had 23.2% fewer offspring (95% CI = (17.4, 28.5%), p < 0.0001), and males from the MA population produced 33.1% fewer offspring than their controls (95% CI = (27.4%, 38.2%), p < 0.0001) (Figure 1). The effects of mutation-accumulation were much less pronounced for females expressing MA-X chromosomes heterozygously. Vials with females expressing heterozygous MA-X chromosomes contained an average of 8.98 offspring (95% CI = (8.57, 9.41)), and females with heterozygous C-X chromosomes produced an average of 9.62 offspring (95% CI = (8.48, 10.10)). Translated to relative fitness heterozygous MA females declined by 6.8% (95% CI = (1.6%, 11.6%), p = 0.01). The relative fitness of males bearing MA-X chromosomes (W_m_) was significantly lower than the relative fitness of homozygous females with the same pool of mutations (W_f_) (mean W_m_/W_f _= 0.87, 95% CI = (0.78, 0.97), p = 0.013), and homozygous MA females had significantly lower relative fitness than their heterozygous counterparts (mean = 0.82, 95% CI = (0.75, 0.90), p = 0.0003).

### Inbreeding depression for female fitness

**Figure 2:**
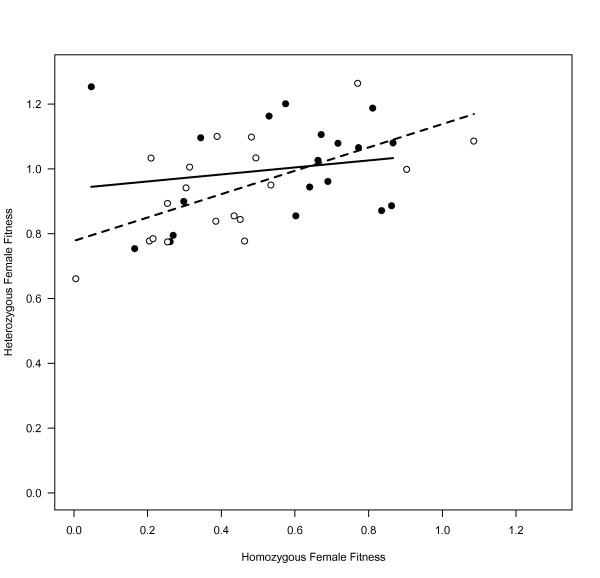
**Correlation between inbred and outbred female fitness**. Outbred and inbred relative fitness were not correlated in the C lines (black circles, solid regression line) and were positively correlated in the MA lines (white circles, dashed regression line), indicating increased dominance of new mutations. Fitness values were calculated relative to the heterozygous control population mean.

Making the X chromosome homozygous had detrimental effects on fitness for females expressing X chromosomes from the both the C and MA populations. For the C-X chromosomes, inbreeding was associated with a 44.1% decline in fitness (95% CI = (40.7%, 47.4%), p < 0.0001). The effect was larger for MA-X chromosomes, where inbreeding was associated with a 54.0% decline in fitness (95% CI = (50.8%, 56.9%), p < 0.0001). For the C lines, there was no correlation between heterozygous and homozygous female fitness (p = 0.46, r^2 ^= 0.16, slope = 0.06). For the MA lines, however, we observed a significant correlation between inbred and outbred line means (p = 0.0048, r^2 ^= 0.38, slope = 0.20) (Figure 2). We tested for a difference in the slope and correlation between the MA and C lines by performing 5,000 bootstrap replicates, which did not reject the null hypothesis for either the slope (p = 0.30) or the correlation (p = 0.21).

### Genetic variation for fitness and heritability

We estimated genetic variance for fitness by fitting a random-effects ANOVA (using line as the only factor) to each treatment/sex combination (Table 1). Significant genetic variance for fitness was found at all levels, and the MA lines had both greater levels of genetic variation and higher heritability than the C lines. CV_E _and CV_P _were corrected for the number of flies in each vial and these corrected estimates were used to infer heritability at the individual-level. This was done by multiplying residual variance estimates by the number of flies in each vial and then subtracting variance due to genotype.

**Table 1:** Heritabity, and coefficients of additive (CV_A_) environmental (CV_E_) and phenotypic (CV_P_) variation for each sex-treatment combination

	h^2^	CV_A_	CV_E_	CV_P_	p-value
C Females (heterozygous)	0.010	0.099	1.00	1.00	0.011
MA Females (heterozygous)	0.023	0.15	0.98	0.99	< 0.0001
C Females (homozygous)	0.089	0.39	1.26	1.32	< 0.0001
MA Females (homozygous)	0.16	0.65	1.49	1.63	< 0.0001
C Males (hemizygous)	0.007	0.12	1.51	1.51	0.041
MA Males (hemizygous)	0.042	0.37	1.76	1.80	< 0.0001

P-values are for the random-effects ANOVA used to estimate variance components

### Intersexual correlations

**Figure 3:**
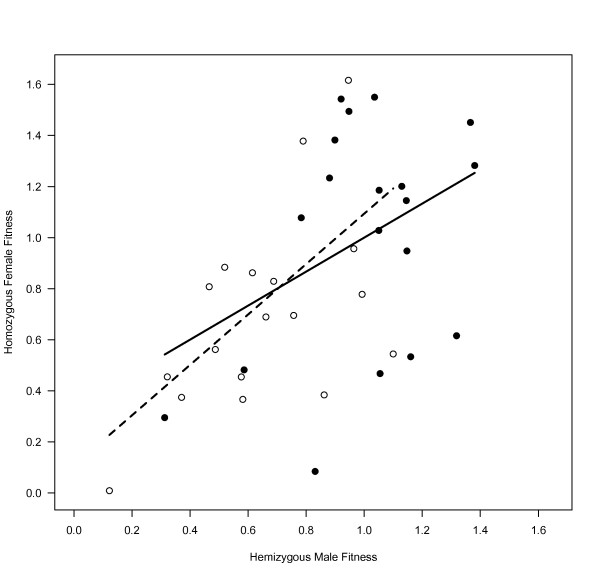
**Correlation between male and homozygous female fitness**. Male and female relative fitness were not correlated in the C lines (black circles, solid regression line) and were significant correlated in the MA lines (white circles, dashed regression line), indicating that new mutations were concordant in the direction of selection. Fitness values were calculated relative to mean control fitness.

We estimated the intersexual genetic correlation for adult fitness in the IV population, for the C- and MA-X chromosomes. For the C lines, we recorded no significant genetic correlation between the line means of females expressing X chromosomes homozygously and the line means of males (p = 0.10, r^2 ^= 0.15, slope = 0.67). In the MA lines, fitness was positively correlated between homozygous MA females and MA males (p = 0.015, r^2 ^= 0.30, slope = 0.99) (Figure 3). We tested for a difference in the slope and correlation between the MA and C lines by performing 5,000 bootstrap replicates, which did not reject the null hypothesis for either the slope (p = 0.48) or the correlation (p = 0.54).

### Estimating mutational effects on fitness

If we assume new mutations interact multiplicatively (i.e., no epistasis), fitness will decline by a fixed percentage each generation, corresponding to a factor  for males and  for females. Based on our observed values of *W_m _*and *W_f _*we estimate this per-generation rate of decline to be 0.53% for females (95% CI = (0.38%, 0.67%)) and 0.80% in males (95% CI = (0.63%, 0.96%)) for the X chromosome. Male fitness thus declined at a rate that was 1.52 times faster than females (95% CI = (1.09, 2.19), p = 0.013). The mean rate of decline for heterozygous female fitness was much lower, at 0.14% (95% CI = (0.03%, 0.25%)).

### Estimating the relative strength of selection on males vs. females (alpha)

Assuming multiplicative effects on fitness and a constant coefficient of selection, the fitness of a genome homozygous for *n *mutations will be:

where *w *= (1 - *s*).

If selection varies across loci, we will have instead, on average:

where  is the geometric mean fitness at individual loci, and  serves as an estimator for the genome-wide selection coefficient ( is not the geometric mean of *s*). The per-generation haploid genomic mutation rate is

The fitness of a genome homozygous for new mutations after *t *generations will then be, on average:

If coefficients of selection vary among loci for males and females, at the *i*^th ^locus we will have:

and we can define:

The fitness of populations of males and females expressing the same pool of mutations after *t *generations can then be expressed as:

Which we can rewrite as

Our estimator for the overall relative strength of selection on males and females () thus also depends on the haploid genomic mutation rate (*U*), which is not known with much precision for any population. For *Drosophila*, experiments typically place the diploid genomic mutations rate (2*U*) in the range of 0.1-1.5 [8,29], which would correspond to a mutation rate on the X chromosome of between 0.01 and 0.15 assuming that mutation rate is uniform across the genome. Using these two extreme values we obtain estimates of  = 1.34 (95% CI = (1.06, 1.77)) and  = 1.50 (95% CI = (1.09, 2.16)), respectively. Thus, variation of over an order of magnitude in *U *has comparatively little effect on our estimate of  given the duration of the MA experiment and the observed values of W_m _and W_f _for this experiment.

### Dominance of new mutations

The dominance of mutations will influence the extent to which population size and structure modulate the consequences of MA. For females, the fitness of heterozygous and homozygous MA populations relative to their controls can be written as

Where  and  represent mean fitness for females after *t *generations of MA relative to their controls in the heterozygous and homozygous states, respectively, and  serves as an estimator for genome-wide dominance coefficient. These equations can be rearranged to give:

As for ,  is not very sensitive to changes in *U *in the range of 0.01-0.15 per X chromosome per generation, given the values of W_m _and W_f _obtained in this experiment (*Ux * = 0.01: = 0.32, 95%CI = (0.08, 0.56), *Ux *= 0.15:  = 0.27, 95%CI = (0.06, 0.51).

## Discussion

Despite decades of research on the properties of new mutations, we know relatively little about their effect on adult fitness. By quantifying the consequences of new X-chromosome mutations for the reproductive success of males and females, we have begun to address this gap. Our results indicate that selection against new mutations differs in magnitude between the sexes, though the direction of change appears broadly concordant. This may be a fundamental feature of many populations due to the ubiquity of sexual selection.

We estimated that the rate of decline in adult fitness due to MA was 0.53% per X chromosome per generation in homozygous females, 0.80% per X chromosome per generation in males, and 0.14% per X chromosome per generation in heterozygous females. Scaled up to the entire haploid genome, this would imply a 2.6% decline in fitness per generation in homozygous females, and a 3.9% decline per generation in homozygous males. The heterozygous female decline is predicted to be much lower, at 0.7% per haploid genome per generation. Thus, the MA treatment was associated with a rapid decline in fitness. Our estimates for the rates of mutational decline in adult fitness are greater than estimates from both classical [30] and recent MA studies using viability [21,31,32]. This is consistent with previous work performed with the *IV *population [33], which found that inbreeding depression for total fitness was mainly due to depression in adult fitness. If the *IV *population is at mutation-selection balance, it is possible that stronger inbreeding depression for adult fitness is reflective of increased mutational pressure. In any case, our results demonstrate that the total mutational load of populations could be much greater than measurements of viability alone would imply.

Potential sources of error in estimating the rate of mutational decline in fitness for a population include confounding factors that bias the rate of mutation-accumulation during MA and factors that bias the measured impact of mutation during fitness assays. We accumulated mutations on hemizygous X chromosomes in *Drosophila *males. In *Drosophila*, the rate of sequence change at neutral sites suggests that the mutation rate is not distinguishable from parity between the sexes [34], so we expect that the baseline rate of sequence change in our study was a fair representation of the normal mutation rate. Because we eliminated sexual selection by passing each MA line through single-X bottlenecks, the opportunity for selection within the MA lines was limited to differences in viability between siblings resulting from a single generation of MA. Under the low competition conditions employed, we expect little viability selection, and even less impact on the rate of mutation-accumulation for genes affecting adult fitness in the MA population.

The control lines were kept as small, effectively asexual populations (*i.e.: *no recombination between X chromosomes within a C-line) to minimize the possibility of adaptation. Adaptation in the control population would artificially inflate the measured decline in fitness due to MA and has been cited as a potentially major source of bias in estimating mutational parameters in other studies [9,35]. Control-X chromosomes were expressed hemizygously and the opportunity for sexual selection existed in these populations; while this, along with a larger population size, will slow down the rate of MA, we cannot eliminate the possibility that some deleterious mutations have fixed in these lines. The presence of mutation-accumulation in the control lines will cause us to underestimate the rate of erosion in fitness due to MA. MA in the control population could also affect our estimates for the relative strength of selection in males and females, if these mutations have sex-specific effects on mean fitness. In particular, mutations in genes with female-limited expression could accumulate freely under our experimental design because C-line chromosomes are only exposed to selection in males. If mutations with larger effects on females had accumulated in the C-lines, this would diminish differences between control and MA females, making the male differential appear larger and inflating . However, there is very little evidence for widespread female-limitation of gene-expression in the *D. melanogaster *genome [36], and the results presented here indicate that most mutations are selected against in both sexes. In addition, direct observations from experiments on the maintenance of control lines over several dozen generations show no evidence of female-specific mutational decline (additional file 1, Figure S1).

Another important consideration stems from the observation that the environmental conditions under which fitness assays are performed can profoundly affect the perceived decline in fitness due to MA. As an example, it is well known that highly competitive conditions exaggerate differences between the control and MA populations: the decline in viability with MA can be nearly 10-fold greater under harsh competitive conditions [21], and diluting the food has also been found to affect the relative performance of MA flies [20]. Many MA studies have used wild-caught or recently domesticated populations; the fitness assays used are unlikely to encapsulate the relevant selective environment. The use of populations in novel environments may also increase the probability of adaptation in the control lines. When measuring the selective effects of mutations in a particular population, it therefore seems sensible to restrict our measures to the conditions that shaped its population-genetic structure. The *IV *population has been maintained under consistent culture conditions for over 700 generations, and we emulated the culture protocol in almost every detail for our fitness assays. We therefore do not anticipate substantial bias in our estimates of fitness decline resulting from the environmental conditions used.

Because we measured the performance of experimental flies as adults by counting their progeny, viability effects may have influenced our measurement of MA for adult fitness. In particular, the offspring of adults from the MA treatment may have suffered in terms of reduced viability, which would inflate our estimate of the effects of MA on adult fitness. This effect would be most pronounced for homozygous MA females, who pass on a full copy of their MA-X chromosomes to their sons, and who may also contribute adverse maternal effects to their offspring. A significant viability effect on the offspring of MA females would make our estimates of  conservative. Strong differences in viability due to MA-X chromosomes should manifest themselves in terms of skewed sex ratios, but we found no differential effect of MA on offspring sex ratio.

We estimated that the magnitude of mutational effects, X-chromosome wide, was approximately 1.4 times stronger in males than in females (1.06-2.14, a range that includes both experimental error and uncertainty around U). In addition, the intersexual genetic correlation for the MA lines was significant and positive. This suggests that, for the majority of new mutations on the X chromosome, selection operates in the same direction for both sexes. This is interesting because the X chromosome is the genomic location most likely to show mutations with sexually antagonistic or sex-independent effects [26,27]. While we do not dispute this, our results nevertheless seem to indicate that most of the mutations we assayed had sexually concordant effects on adult fitness. However, even if all mutations had concordant effects on fitness, the range in  presented here (1.06-2.14) could be consistent with anything from modest benefits of sexual selection to females to a greater than two-fold fitness advantage compared to a hypothetical asexual competitor, depending on the mutation rate, so refining estimates for  through further study will be critical.

We found that the effects of new mutations were positively correlated between males and females when females expressed MA-X chromosomes homozygously, but new mutations will most often be expressed heterozygously in females. In the MA lines, we found a positive association between homozygous and heterozygous female fitness values suggesting that new mutations are partially additive. We estimate that the population-wide dominance coefficient for new mutations is about 0.3. As new mutations will be expressed hemizygously in males and heterozygously in females, the effectiveness of selection will be much greater for males. Based on the rate of heterozygous female decline in fitness, we estimate the 'effective'  to be greater than 5 for the X chromosome.

The hemizygosity of males should result in more efficient selection on recessive alleles on the X chromosome. When these alleles have concordant directional effects across the sexes, this will result in reduced genetic load, an expectation corroborated by the absence of detectable inbreeding depression for juvenile viability in several populations of *Drosophila melanogaster *[33,37]. For adult fitness, however, our results indicate that there remains substantial standing deleterious genetic variation on the X chromosome, as evidenced by the presence of substantial inbreeding depression for female fitness in the control group.

There are several possible explanations for the high genetic load for adult fitness found on the X chromosomes of the *IV *population. First, adult fitness might represent a larger mutational target than juvenile viability. We believe this is likely because adult fitness will be influenced both by juvenile traits not captured by viability (for example larval condition upon pupation) and by mate competition. Second, sexually antagonistic alleles, though in the minority, may nonetheless exert considerable effects on net fitness because the genetic load associated with them tends to be greater than for concordantly selected alleles [38]. In a separate population of Drosophila (LH_m_), the amount of sexually antagonistic variation on the X chromosome was sufficient to cause a negative intersexual genetic correlation for adult fitness [28]. In that study, the X chromosome was estimated to account for about 45% of the total genetic variation in fitness, and nearly all of the sexually antagonistic variation. Even so, most new mutations in the LH_m _population are predicted to be under concordant selection [39], although the intersexual genetic correlation for fitness was not measured.

Similarly, the X chromosome appears to harbour a disproportionate amount of fitness variation in the *IV *population. Previous work with the *IV *population suggested that completely inbred *IV*-derived females were 36% as fit as their outbred counterparts [33]. In this study, females inbred for the X chromosome were 57% as fit as outbred females. Assuming multiplicative fitness effects, we infer that females completely inbred for the autosomes would be 63% as fit as outbred females. The X chromosome therefore seems to contribute more than half of the total inbreeding depression for adult female fitness, despite accounting for only a fifth of the gene content. The presence of segregating sexually antagonistic alleles on the X chromosomes in the *IV *population would be consistent with the disproportionate amount of genetic load on this chromosome, and is supported by a lack of positive intersexual correlation in the control lines.

Moderate amounts of sexually antagonistic variation have the potential to reduce the benefits of sexual selection, but the extent to which it does so depends on the relative amount and intensity of sexually concordant and sex-specific selection on the genomic scale [38]. Our results suggest that, at least for the X chromosome, the majority of mutations have sexually concordant effects, as our estimate of  is a global estimate. As long as the fraction of sexually antagonistic alleles generated by mutation is small, our results suggest that sexual selection could still yield net benefits to females, though estimating the precise fraction of mutations that are concordantly selected vs. sexually antagonistic should be a priority.

No study yet designed has been able to estimate all of the relevant properties of new mutations. Molecular methods are increasingly being used [29], but can only directly measure the total rate of sequence change; without contemporary fitness data these methods provide only indirect estimates of the deleterious mutation rate. Conversely, fitness estimates on their own, while providing important insight into the consequences and character of new mutations, do not produce reliable estimates of the mutation rate. Mutation-accumulation in well-defined and replicable experimental populations such as the *IV *population, combined with advances in sequencing, could provide a much clearer picture of the fate of new mutations in populations than either technique in isolation. For example, one approach could involve sequencing MA lines to obtain the actual changes having occurred during MA, and then allowing replicated populations to purge these mutations in the standard laboratory environment. The rate at which mutations are eliminated would permit estimation of the strength of selection against them.

## Conclusions

Our data highlight the importance of quantifying adult fitness and incorporating the distinctive features of X-linkage to understanding the consequences of mutation. Erosion of adult fitness due to MA on the X-chromosome was high, and the finding of sex-specificity in the strength of selection against deleterious mutations adds a new dimension to the problem of the maintenance of sexual reproduction. Direct estimates of the deleterious mutation rate, the range of variation in alpha between mutations, and the fraction of sexually antagonistic mutations will be important in quantifying the net cost/benefit of sexual reproduction for populations. Our data represent a critical first step in this direction: they suggest that most mutations are concordantly selected in the two sexes, and so the potential exists for female fitness to improve as the result of selection on males.

## Methods

### Stocks and culture conditions

All flies sampled for this experiment were derived from the same lab population - the *Ives *(*IV*) population. This lab-based population was established from a wild-caught sample of 200 females and 200 males in Amherst, Massachusetts in 1975 [39]. From 1981 onwards, the *IV *laboratory population has been maintained as a large outbred stock at a minimum population size of 1000 individuals at 25°C, 50% relative humidity, on a 14 day, discrete generation cycle with moderate densities of 60-120 individuals per vial with 10 mL of banana/agar/killed-yeast medium [40]. On Day 14, the population is placed under CO_2 _anaesthesia, mixed and redistributed into new vials to oviposit until ~100 eggs are laid in each vial. This usually takes approximately 30 minutes, and represents the only opportunity for offspring production.

The *IV_bw _*population, which was created by backcrossing a recessive brown-eye colour marker (*bw*^1^) into the *IV *population, served as an outbred, genetically similar population for use as competitors against *IV *flies for measurements of fitness. This population is maintained under a culture protocol identical to the *IV *population, and is periodically backcrossed to the *IV *population to prevent drift between the focal and competitor populations.

Two additional stocks were created in order to express X chromosomes of interest in males and females. The *DX-IV *population is a copy of the *IV *population into which a compound X-chromosome (C(1)DX *y f*) has been introgressed. This compound-X (DX) chromosome forces the normal pattern of sex-chromosome inheritance to be reversed: males crossed with DX-bearing females pass on their X chromosome to their sons, and receive a Y chromosome from their mother. The *FM*-*IV *population is a copy of the *IV *population into which an X-balancer chromosome was introgressed (FM7a).

### Mutation-accumulation protocol

A sample of 19 genetically variable X chromosomes from the *IV *population was obtained by singly crossing males from the *IV *population to virgin females bearing DX chromosomes. Males from these crosses were fixed for the X chromosomes of their fathers and used to simultaneously found two initially identical groups.

**Figure 4:**
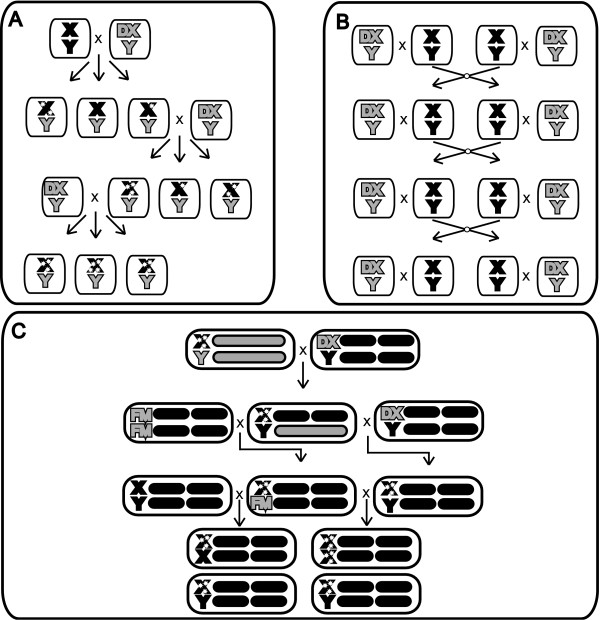
**Generation of mutation-accumulation lines and experimental flies**. (A) Mutation-accumulation protocol. A single male bearing an IV-derived X chromosome was mated to multiple DX (C(1)DX *y f*)-bearing females. A single son, bearing new mutations (white stars), was randomly selected to found the next MA generation. Each generation, triplicate crosses were performed to guard against line loss. (B) Maintenance of control lines. Control lines were initially founded from the same X chromosomes used to create the MA lines. Each generation, males from two vials were mixed together and then split into two vials, each containing 8-10 males and 16-20 DX bearing females. (C) Generation of experimental MA flies. The autosomes from the C and MA males were substituted with a set of marked translocated autosomes ((T(2 : 3)*rdgc st in ri p^p ^bw*) (grey bars) and crossed to *DX-IV *females. The resulting males were subsequently crossed to both *DX-IV *females and *FM *(FM7a)*-IV *females to yield males fixed for the MA-X chromosome and females with a balanced MA-X chromosome. Females carrying a balanced C or MA X chromosome in the *IV *autosomal background were crossed to either random *IV *males to generate heterozygous females or were crossed to males bearing MA X-chromosomes to generate homozygous females. Males bearing MA chromosomes were collected from both crosses. The sequence of crosses to generate control experimental flies was identical.

For the mutation-accumulation (MA) population, each of the 19 lines was taken through a single-X bottleneck every generation (Figure 4A). To accomplish this, three males descended from the same father were separately mated to groups of five virgin DX-bearing females to prevent line loss. These groups were housed in 'conditioning vials' with supplemental live yeast for two days, after which they were transferred to fresh vials and allowed to oviposit overnight. The conditioning vials were kept and used as a backup in case of failure in the oviposition vials. The oviposition vials were reared under reduced density (approximately 40 individuals) to minimize competition. After twelve days, three males were again chosen from a single vial to start the next cycle, so that for each generation of MA all of the males selected descended from the same father. By creating a single X-chromosome bottleneck each generation, selection on new germ-line mutations was minimized, except against those mutations causing death or sterility in males.

The control (C) lines were maintained in the same fashion as the MA lines except that each X-chromosome line was maintained by crossing 8-12 males and 16-20 females in two vials (16-24 males per population), keeping rearing densities at approximately 100 individuals and mixing between vials each generation (Figure 4B). By maintaining the C lines in relatively small populations without recombination we hoped to prevent the possibility of adaptation in the control lines, a problem that has plagued the interpretation previous MA studies [9,35], while allowing for sufficient selection to prevent significant depression in fitness due to MA. This method of maintaining control lines is likely to be ineffective in preventing mutations of very small effect to fix, however, and will make our estimates of the total effect of MA conservative.

### Creation of experimental lines

For fitness assays, X chromosomes from the C and MA lines were placed in a random outbred *IV *autosomal background (Figure 4C). The autosomes from the C and MA males were first substituted with a set of marked translocated autosomes ((T(2: 3)*rdgc st in ri p^p ^bw*). Eight to ten C or MA males from each line were then crossed to virgin *DX-IV *females. Males from this cross carried X-chromosomes from their parent lines, an *IV *Y-chromosome from the *DX-IV *females, a set of translocated autosomes, a set of random *IV*-derived autosomes, and were subsequently crossed to both *DX-IV *females and *FM-IV *females. Males from the *DX-IV *cross were fixed for the X chromosome of interest and possessed a wild-type set of autosomes, while females from the *FM-IV *cross carried balanced X chromosomes along with a set of random *IV *autosomes.

Virgin females carrying a balanced C or MA X chromosome in the *IV *autosomal background were crossed to either random *IV *males to generate heterozygous-X females or were crossed to males bearing C and MA X-chromosomes in an *IV *autosomal background to generate homozygous-X females. Males bearing the C and MA chromosomes in an *IV *autosomal background were collected from both crosses. Both heterozygous and homozygous females were therefore produced from the same maternal genotype, to remove the possibility of confounding maternal effects. The normal pattern of sex-chromosome inheritance was also preserved in the production of experimental flies.

### Fitness Assay

The effects of mutation are known to change as a result of both the physical environment and the genetic environment [20,21]. The *IV *population-genetic structure has been shaped by virtually unchanging selection pressure for over 700 generations: the effects of mutations in this genetic background are therefore best interpreted in the environment to which the population has adapted. Our measure of adult fitness was designed to capture the outcome of adult competition under *IV *culture conditions, while making the results of such competition tractable.

We transplanted experimental flies from the C or MA lines using light CO_2 _anesthesia during the period of peak adult eclosion (Day 9 post-oviposition) in same-sex groups of 5 to an age-synchronized culture of IV*_bw _*reared under standard conditions. For five days, the experimental flies were allowed to acquire resources and mates in the competition vials. On Day 14 each vial was individually subjected to 2.5 minutes of CO_2_, to simulate the amount of gas normally received when *IV *vials are mixed, and placed into vials containing fresh medium for oviposition to standard culture densities (25-30 minutes). The adults were then removed from the vials and the sex/number of progeny from the target individuals (distinguishable by their red eyes) was scored twelve to fourteen days later, sufficient time for all of the adults to emerge. The number of progeny present in the vials measures the success of their parents in the previous generation. There will also be an influence of juvenile viability, but this will make our results conservative with respect to  (see Discussion). Each treatment/line/sex combination was replicated 20 times for a total of 2,280 vials.

### Statistical Analysis

Parameter estimates were derived using the normalized likelihood method [40], using the R statistical package [41]. Normalized likelihoods satisfy frequentist principles of inference but are also equivalent to Bayesian analyses using flat priors [42,44]. The normalized likelihood distribution of a parameter *θ *given data *Y *is equal to:

Where the denominator is simply a normalizing constant such that the likelihood distribution has unit area (or sum, in the discrete case). The subsequent posterior distribution (or likelihood density) can be used for point and interval estimation of *θ*, and numerical methods readily yield estimates for various functions of *θ*. Where the likelihood function also depends on other parameters (for example, the likelihood for the mean in a normal distribution also depends on the standard deviation), we take the marginal likelihood taken over all values of the second parameter.

We calculated the posteriors for the rate of red-eyed offspring production (number of red-eyed offspring produced in the oviposition period) for each line/sex/treatment combination using the Negative-Binomial likelihood function.

Where the *y_i _*are the numbers of red-eyed offspring in each vial of a particular line/sex/treatment combination *ϕ*, is a dispersion parameter and *p *is the probability of success, such that  and *λ *is the mean offspring production for those flies. We took the exponent of the log-likelihood function to simplify calculation. At high values of *ϕ*, the negative-binomial distribution approaches a Poisson distribution with mean *λ*. We estimated *ϕ *separately for males and females, because exploratory analysis suggested that the male data was more dispersed than the female data.

We then evaluated the likelihood function at 5 000 × 5 000 grid spanning a large interval of *λ*(10^-10 ^≤ *λ *≤ 30) and *ϕ*(10^-10 ^≤ *ϕ *≤ 100) prior to normalization and marginalization in order to obtain accurate posteriors. Because the dispersion parameter is strictly positive and the resulting distribution asymptotically approaches a Poisson distribution at high values, we will tend to over-estimate overdispersion by cutting off the likelihood surface at 100 (this was done for computational reasons). If the true distribution is Poisson-distributed, our confidence intervals will be somewhat wider, and our p-values will be conservative.

We estimated parameters depending on multiple line means (for example, the group MA male mean) by numerical methods. For each line we first sampled 10 000 means according to their posterior probabilities and then combined them according to the desired function of the *λ*. For example, the point estimate and 95% confidence interval for the group MA male mean was calculated by taking 5 000 averages of the 19 MA male line means, where each MA line mean is a randomly sampled value from the posterior distribution for that line. The mean and 95% confidence interval of the resulting distribution corresponds to the point estimate and 95% confidence interval for the group MA male mean. P-values were estimated in a similar fashion, by calculating the area of the empirical distribution corresponding to the desired test. The main advantage of this method is the relative ease with which point and interval estimates for parameters that are complicated functions of the data (for example,  and ) can be obtained, without having to first derive the appropriate sampling distribution.

## Authors' contributions

MM created the MA lines, carried out the experiments, analyzed the data, and drafted the manuscript. JB participated in the design and execution of the experiment, and helped edit the manuscript. CMK participated in the maintenance of the MA lines, the execution of the experiment, and helped edit the manuscript. AKC helped conceive the study and MA protocol, and helped edit the manuscript. All authors have read and approved the final manuscript.

## Supplementary Material

Additional file 1 **Figure S1**. Relative fitness of whole-genome control lines, expressed as both females (red) and males (blue) over several generations of maintenance according to the protocol described in the Methods. Mean fitness of each point represents fitness of control populations, relative to the most fit control line within each sex/assay. The estimates for g5 and g32 come from a separate set of lines than those used for the estimates at g12, g18, g25, g35, and g50. The slope of the regression was not significant when the control genomes were expressed either as females (slope = -.003, R2 = 0.27, p = 0.225) or as males (slope = -.004, R2 = 0.12, p = 0.23). Click here for file
