# Supplementary figures and images for: Experimental mutation-accumulation on the X chromosome of Drosophila melanogaster reveals stronger selection on males than females
Source: BMC Evol Biol. 2011 Jun 6;11:156. doi: 10.1186/1471-2148-11-156 (PMC3134001; doi:10.1186/1471-2148-11-156)

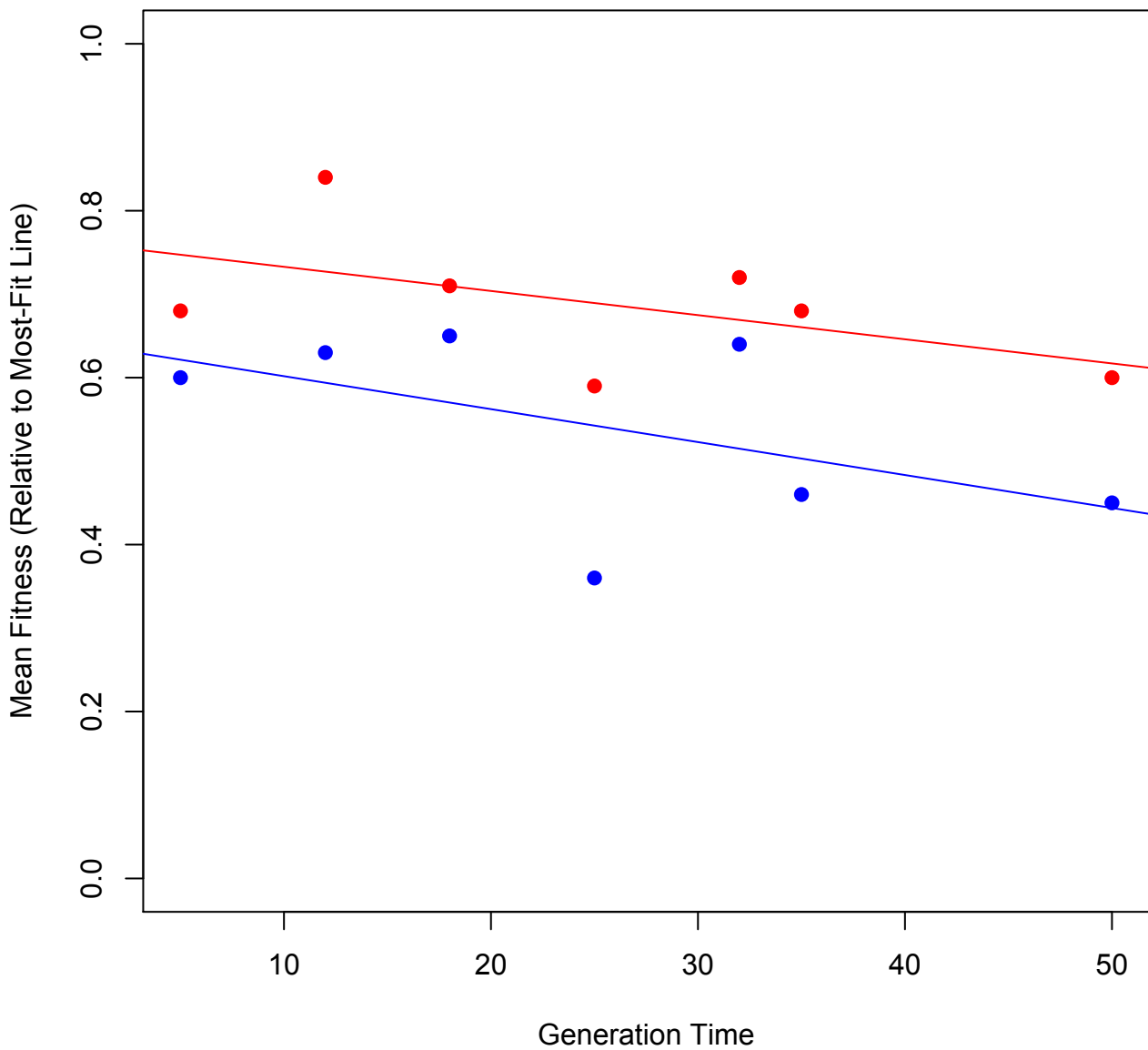

Supplement: Additional file 1 — Figure S1. Relative fitness of whole-genome control lines, expressed as both females (red) and males (blue) over several generations of maintenance according to the protocol described in the Methods. Mean fitness of each point represents fitness of control populations, relative to the most fit control line within each sex/assay. The estimates for g5 and g32 come from a separate set of lines than those used for the estimates at g12, g18, g25, g35, and g50. The slope of the regression was not significant when the control genomes were expressed either as females (slope = -.003, R2 = 0.27, p = 0.225) or as males (slope = -.004, R2 = 0.12, p = 0.23). [file 1471-2148-11-156-S1.PDF]
